# Dynamic causal modelling of eye movements during pursuit: Confirming precision-encoding in V1 using MEG

**DOI:** 10.1016/j.neuroimage.2016.02.055

**Published:** 2016-05-15

**Authors:** Rick A. Adams, Markus Bauer, Dimitris Pinotsis, Karl J. Friston

**Affiliations:** a The Wellcome Trust Centre for Neuroimaging, Institute of Neurology, University College London, 12 Queen Square, London WC1N 3BG, UK; b School of Psychology, University Park, Nottingham University, Nottingham, NG7 2RD, UK

**Keywords:** Oculomotor control, Smooth pursuit, Visual occlusion, Active inference, Dynamic causal modelling, Magnetoencephalography, Precision

## Abstract

This paper shows that it is possible to estimate the subjective precision (inverse variance) of Bayesian beliefs during oculomotor pursuit. Subjects viewed a sinusoidal target, with or without random fluctuations in its motion. Eye trajectories and magnetoencephalographic (MEG) data were recorded concurrently. The target was periodically occluded, such that its reappearance caused a visual evoked response field (ERF). Dynamic causal modelling (DCM) was used to fit models of eye trajectories and the ERFs. The DCM for pursuit was based on predictive coding and active inference, and predicts subjects' eye movements based on their (subjective) Bayesian beliefs about target (and eye) motion. The precisions of these hierarchical beliefs can be inferred from behavioural (pursuit) data. The DCM for MEG data used an established biophysical model of neuronal activity that includes parameters for the gain of superficial pyramidal cells, which is thought to encode precision at the neuronal level. Previous studies (using DCM of pursuit data) suggest that noisy target motion increases subjective precision at the sensory level: i.e., subjects attend more to the target's sensory attributes. We compared (noisy motion-induced) changes in the synaptic gain based on the modelling of MEG data to changes in subjective precision estimated using the pursuit data. We demonstrate that imprecise target motion increases the gain of superficial pyramidal cells in V1 (across subjects). Furthermore, increases in sensory precision – inferred by our behavioural DCM – correlate with the increase in gain in V1, across subjects. This is a step towards a fully integrated model of brain computations, cortical responses and behaviour that may provide a useful clinical tool in conditions like schizophrenia.

## Highlights

• The brain encodes states of the world probabilistically with means and precisions.
• Precision (inverse variance) may be encoded by the synaptic gain of pyramidal cells.
• We estimate subjects' sensory precision using a model of oculomotor pursuit and DCM.
• We estimate subjects' synaptic gain in V1 using DCM of MEG data during pursuit.
• Estimates of synaptic gain in V1 and sensory precision are significantly correlated.

## Introduction

In recent work (Adams et al., 2015), we used a generative model of oculomotor pursuit based on predictive coding and active inference – a Bayes-optimal formulation of action and perception – to predict the eye movements of subjects viewing targets whose velocities vary in precision (inverse variance). This dynamic causal modelling (DCM) of behaviour provides estimates of (subjective) precision that subjects adopt in their hierarchical models of sensory input. From these subjective precisions we derived hypotheses about the gain of neurons that are thought to encode precision — which we test using biophysical DCM of magnetoencephalography (MEG) in this paper.

Our aim is to develop empirical tools that can predict and quantify the precision of – or confidence in – beliefs subjects entertain about the causes of their sensations. A major goal for neuroscience is to validate formal models of a particular task that can predict not just computational processes, but also the physiological basis of those processes (as measured using brain imaging techniques) and their behavioural results, e.g. choices or movements. Before integrating behavioural and imaging models, one must establish that they make similar inferences about physiological and behavioural responses. Here, we establish this sort of construct validity using behavioural and biophysical DCMs of pursuit and MEG data, respectively.

Using a hierarchical model of visual pursuit, we showed that characteristic schizophrenic pursuit deficits – e.g. pronounced slowing during target occlusion – could be reproduced by decreasing high-level precision in the model (Adams et al., 2012). As the ‘gain’ of oculomotor pursuit has been shown to be inversely proportional to the size of random fluctuations around target velocity (Tavassoli and Ringach, 2009), we subsequently tried to *induce* a loss of high-level precision in normal subjects by adding random fluctuations to the velocity of a sinusoidal target (Adams et al., 2015). We quantified changes in subjective precisions using our pursuit model. We discovered that – contrary to our expectations – subjects (on average) responded to the noisier target not by reducing their high-level precision but by increasing their (low level) sensory precision. If one equates the optimisation of precision in hierarchical predictive coding with ‘attention’ (Feldman and Friston, 2010, Moran et al., 2013), this means that, in effect, subjects attended more to the sensory attributes of the target in the noisy condition.

In this study, we sought to replicate and extend these findings by repeating our experiment with concurrent MEG data acquisition. Our four predictions (the first two being replications, the second two hypotheses) were that decreasing the precision (increasing the variance) of target velocity would:

1. increase the lag of the eye behind the target when the latter is visible, and reduce anticipatory saccadic movements when it is occluded (replicating our earlier finding);
2. increase sensory precision, as inferred from eye trajectories under a behavioural DCM (replicating our earlier finding);
3. reduce the self inhibition (i.e. increase the gain) of superficial pyramidal cells at the sensory level (i.e. V1/V2) – a neurobiological correlate of increasing sensory precision – under a biophysical DCM of evoked MEG data.
4. Finally, given that both the behavioural and physiological DCMs report the same subjective precision, the corresponding estimates (of changes in precision and gain) should correlate over subjects.

Evoked rather than induced responses (e.g. Dunkley et al., 2011) were analysed, because we wanted to assess the transient responses in early sensory areas to visual input.

## Materials and methods

### Active inference and behavioural modelling

Here, we outline the active inference framework and pursuit modelling: details of the former can be found in Friston et al. (2010) and further explication of the latter in Adams et al. (2015). The relationships among hidden causes and states (in the real world), internal states of the nervous system, and their coupling via sensation and action can be summarised formally as follows:

1 $$\begin{array}{c} s=g\left(x,v,a\right)+\omega_{s}\\ \dot{x}=f\left(x,v,a\right)+\omega_{x}\\ \dot{a}\left(\tilde{s}\right)=-\partial_{a}F\left(\tilde{s},\tilde{\mu }\right)\\ \dot{\tilde{\mu }}=D\tilde{\mu }-\partial_{\tilde{\mu }}F\left(\tilde{s},\tilde{\mu }\right) \end{array}$$

Here, real-world states are in boldface, while the states of the agent are in italics. The agent's sensory states *s* are a function **g** of hidden states **x** and causes **v** and action *a*, and some random fluctuations **ω**_*s*_. The dynamics of states in the real world **ẋ** themselves evolve in a similar fashion.

The lower equations come from the free energy principle (Friston et al., 2006), under which the internal states of the nervous system $\tilde{\mu }$ (including their higher order derivatives, denoted by ~) change in order to minimise the variational free energy *F* of internal states and sensations (D is a differential operator returning generalised motion, such that $D\tilde{\mu }=\left(\mu^{\prime },\mu^{\prime \prime },\mu^{\prime \prime \prime },\ldots \right)$). These internal states correspond to expectations about states of the world. Minimising free energy means that the brain adjusts its expectations to maximise the evidence for its (generative) model of sensory inputs (Ballard et al., 1983, Bialek et al., 2001, Dayan et al., 1995, Gregory, 1980, Grossberg et al., 1997, Knill and Pouget, 2004, Olshausen and Field, 1996). This is just a formal expression of the Bayesian brain hypothesis (Maloney and Zhang, 2010, Weiss et al., 2002, Yuille and Kersten, 2006). The minimisation of free energy usually corresponds to minimising prediction error under a hierarchical model of sensory input; i.e., the process of perception. Action *a* also minimises free energy by changing the sensory states sampled according to the predictions of the generative model (Friston et al., 2010). Note that action $a\left(\tilde{s}\right)$ is function of sensory (proprioceptive) states and their predictions — and is a key variable in closing the action and perception cycle. In short, both perception and action minimise free energy or, more simply, prediction errors.

The modelling in this study can be regarded as ‘meta-Bayesian’ because we are using Bayes' rule twice: first, we assume that our subjects engage in active Bayesian inference using a likelihood model of their sensations *p*(*s*|*θ*_*s*_, *m*_*s*_), with parameters *θ*_*s*_ , about which they have prior beliefs *p*(*θ*_*s*_|*η*, *m*_*s*_) with sufficient statistics *η*. We then estimate their prior beliefs using Bayes rule, by assuming action maximises model evidence (third equality above). This *subjective model* is absorbed into an *objective model p*(*a*|*η*, *θ*_*o*_, *m*_*o*_) of their responses (Daunizeau et al., 2010). This enables one to estimate subjective priors (e.g. the precision) given observed behaviour. This dual use of Bayes' rule can be summarised as:

2 $$\begin{array}{c} \mathrm{subjective}\;\mathrm{model}\;\left(m_{s}\right)\left\{\begin{array}{l} p\left(\theta_{s}|s,\eta ,m_{s}\right)=\frac{p\left(s|\theta_{s},m_{s}\right)p\left(\theta_{s}|\eta ,m_{s}\right)}{p\left(s|\eta ,m_{s}\right)}\\ F\left(\tilde{s},\tilde{\mu },\eta \right)\approx -\ln p\left(s|\eta ,m_{s}\right) \end{array}\right)\\ \mathrm{objective}\;\mathrm{model}\;\left(m_{o}\right)\left\{\begin{array}{c} p\left(\eta ,\theta_{o}|a,m_{o}\right)=\frac{p\left(a|a{\left(\eta \right)}^{*},\theta_{o},m_{o}\right)p\left(\eta ,\theta_{o}|m_{o}\right)}{p\left(a|m_{o}\right)}\\ \dot{a}{\left(\eta \right)}^{*}=-\partial_{a}F\left(\tilde{s},\tilde{\mu },\eta \right)\\ \dot{\tilde{\mu }}\left(\eta \right)=D\tilde{\mu }-\partial_{\tilde{\mu }}F\left(\tilde{s},\tilde{\mu },\eta \right) \end{array}\right) \end{array}$$

In brief, this meta-Bayesian approach assumes subjects minimise (a free energy bound on) the marginal likelihood of their sensory observations through action. This is known as active inference. Active inference assumes that the subject's generative model is nonlinear and dynamic, with a hierarchical structure in which the output of one level (*i*) constitutes the input to the next:

3 $$\begin{array}{c} s=g^{\left(1\right)}\left(x^{\left(1\right)},v^{\left(1\right)}\right)+\omega_{v}^{\left(1\right)}\\ {\dot{x}}^{\left(1\right)}=f^{\left(1\right)}\left(x^{\left(1\right)},v^{\left(1\right)}\right)+\omega_{x}^{\left(1\right)}\\ \vdots \\ v^{\left(i-1\right)}=g^{\left(i\right)}\left(x^{\left(i\right)},v^{\left(i\right)}\right)+\omega_{v}^{\left(i\right)}\\ {\dot{x}}^{\left(i\right)}=f^{\left(i\right)}\left(x^{\left(i\right)},v^{\left(i\right)}\right)+\omega_{x}^{\left(i\right)}\\ \vdots \end{array}$$

This generative model specifies a probability density function over sensory inputs and hidden states and causes (*x*^(*i*)^, *v*^(*i*)^) ∈ *θ*_*s*_ that defines the free energy $F\left(\tilde{s}\right)\approx -\ln p\left(\tilde{s}|m_{s}\right)$. The model assumes Gaussian random fluctuations (*ω*_*x*_^(*i*)^, *ω*_*v*_^(*i*)^) on the motion of hidden states and causes, which play the role of sensory noise at the first level and induce uncertainty about states at higher levels. The (inverse) amplitudes of these fluctuations are quantified by their precisions (*Π*_*x*_^(*i*)^, *Π*_*v*_^(*i*)^) ∈ *θ*_*s*_.

In terms of the biological implementation of active inference, expectations can be updated using predictive coding (Friston, 2005, Rao and Ballard, 1999). This involves expressing free energy in terms of prediction errors and then associating predictions and prediction errors with various neuronal populations in the cortical laminae — such that superficial pyramidal cells pass ascending prediction errors to higher hierarchical levels and receive descending predictions from deep pyramidal cells (Mumford, 1992). In this setting, precision is encoded by the postsynaptic gain of cells reporting prediction error; i.e., the gain of pyramidal cells sending forward connections in the brain (Feldman and Friston, 2010). One can regard prediction errors as reporting what is newsworthy (what cannot be predicted), while expected or subjective precision turns up the ‘volume’ of processing channels with more reliable news.

In the active inference framework, action is produced by proprioceptive predictions that descend to the level of (pontine) cranial nerve nuclei and the spinal-cord. In the oculomotor system, proprioceptive predictions are likely transformed into motor commands by a simple inverse model (rather than classical reflexes, as in other motor systems). Note that the only way that action can minimize free energy is to change sensory (proprioceptive) prediction error, enabling action to fulfil predictions about (hidden) states of the world: see Adams et al. (2013a) and Friston et al. (2010) for details.

In summary, we have a formulation of perception and action based on Bayes-optimal exchanges with the world and a generative model. To use this formulation in any particular setting, one has to specify the particular generative model in Eq. (3). We now turn to the pursuit model used in this work.

### Oculomotor pursuit model

This oculomotor pursuit model generates the pursuit of a smoothly moving target that accommodates the synergy between the pursuit and saccadic systems (Orban de Xivry et al., 2006). It is neither a model of smooth pursuit per se, nor of separate (pursuit and saccadic) systems: it does not differentiate between pursuit and saccadic movements because it is modelling grand-averaged eye displacements. The purpose of this model is to derive estimates of subjective precision at different levels in a hierarchical model of pursuit movements. A full account of this model can be found elsewhere (Adams et al., 2015); the following is a summary.

**Fig. 1:**
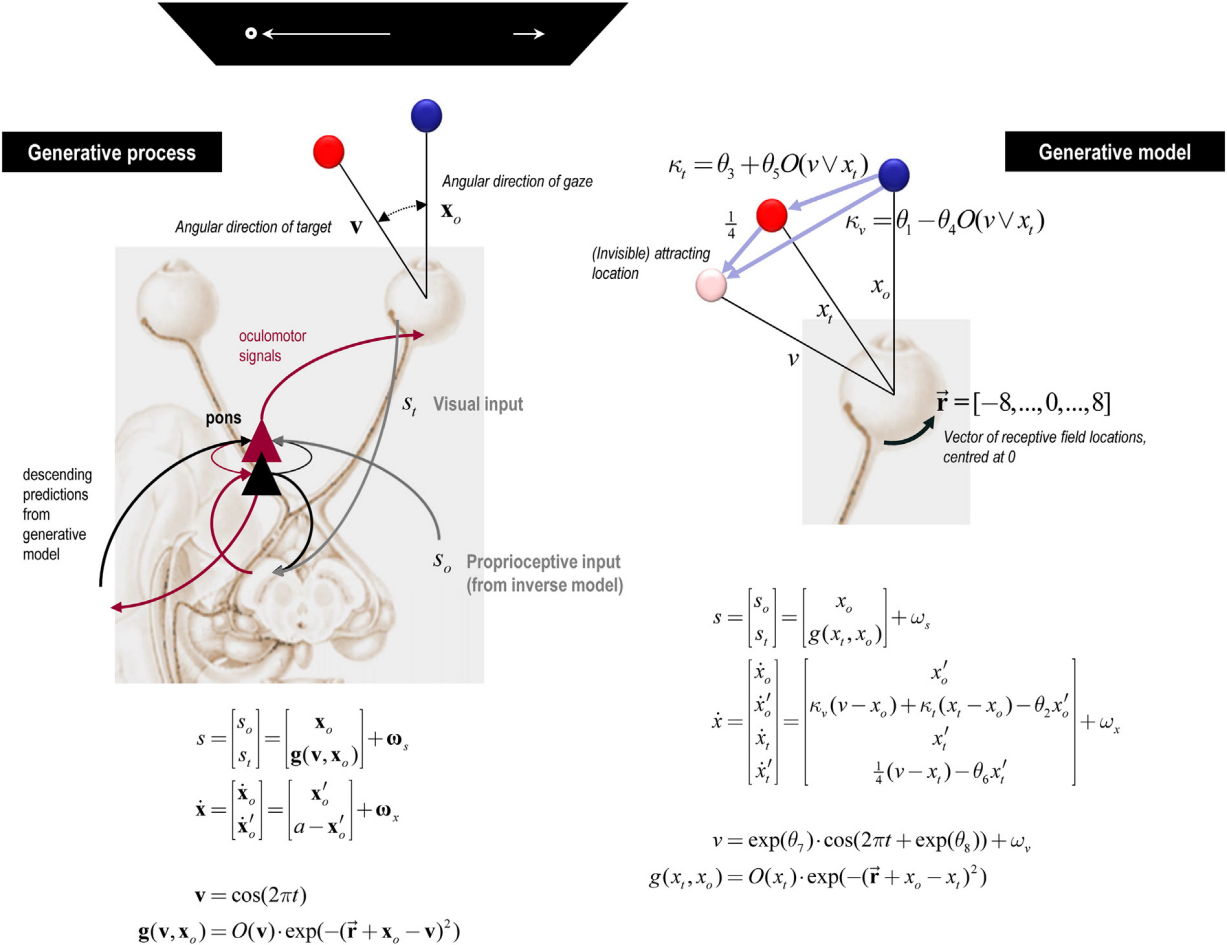
Generative process and generative model used to simulate oculomotor pursuit. This schematic illustrates the process (left panel) and generative model of that process (right panel) used to simulate Bayes-optimal pursuit of a target moving sinusoidally along a horizontal path, part of which is occluded (top left). The graphics on the left show part of a putative predictive coding scheme (with superficial pyramidal cells in red and deep pyramidal cells in black in the pontine nuclei) processing proprioceptive information during smooth pursuit. These cells receive proprioceptive information from an inverse model in the subcortical oculomotor system and respond reflexively to minimise proprioceptive prediction error through action. This prediction error rests on descending predictions from the generative model on the right. The actual movement of the target is determined by a hidden cause (target location), which determines the visual input for any given direction of gaze (equations on the left). *O* is an occluder function whose output is 0 when the target location **v** is occluded and 1 otherwise. The generative model entails beliefs about how the target and eyes move (equations on the right). The agent believes both the target and centre of gaze are drawn to a (fictive) attracting location *v* that is a sinusoidal function of time with parameters controlling its amplitude and phase (*θ*_7_, *θ*_8_). This location attracts the target with a viscosity *θ*_6_. Changes in eye velocity *ẋ*_*o*_′ are determined by a weighted combination of the distance between the eye and the invisible location and target *κ*_*v*_(*v* − *x*_*o*_) + *κ*_*t*_(*x*_*t*_ − *x*_*o*_). Each weight (*κ*_*v*_, *κ*_*t*_) has a fixed component and an occluder-dependent component (c.f. Bogadhi et al., 2013) that depends on the remaining kinetic parameters (*θ*_1_, *θ*_3_, *θ*_4_, *θ*_5_), where the viscosity of eye movements is encoded by *θ*_2_. Real states that are hidden from observation in the real world are in bold, whereas the hidden states assumed by the generative model are in italics. Please see Adams et al. (2015) – from which this figure is adapted – for a full description of the variables and equations.

The ‘real world’ process generating sensory input is modelled by Eq. (4) (see also Fig. 1, left side):

4 $$\begin{array}{c} s=\left[\begin{array}{c} s_{o}\\ s_{t} \end{array}\right]=\left[\begin{array}{c} x_{o}\\ g\left(v,x_{o}\right) \end{array}\right]+\omega_{s}\\ \dot{x}=\left[\begin{array}{c} {\dot{x}}_{o}\\ {\dot{x}}_{o}^{\prime } \end{array}\right]=\left[\begin{array}{c} x_{o}^{\prime }\\ a-x_{o}^{\prime } \end{array}\right]+\omega_{x}\\ v=\cos\left(2\pi t\right)\\ g\left(v,x_{o}\right)=O\left(v\right)\cdot \exp\left(-{\left(\vec{r}+x_{o}-v\right)}^{2}\right) \end{array}$$

The world provides sensory input in two modalities: the horizontal angular displacement of the eye *s*_*o*_ and the angular position of the target on the retina *s*_*t*_. The former corresponds to the angular direction of gaze **x**_*o*_ and the latter is generated by the angle between gaze and target **x**_*o*_ − **v** that determines which of 17 receptive fields in vector $\vec{r}$ becomes active. The retinal input is affected by an occluder function of target location *O*(**v**) that reduces it to zero whenever the sinusoidally-varying target location is behind the occluder. The hidden states of the model are oculomotor angle and velocity (**x**_*o*_, **x**_*o*_′) where velocity is driven by action.

The generative model of this process is modelled by Eq. (5) (see also Fig. 1, right side):

5 $$\begin{array}{c} s=\left[\begin{array}{c} s_{o}\\ s_{t} \end{array}\right]=\left[\begin{array}{c} x_{o}\\ g\left(x_{t},x_{o}\right) \end{array}\right]+\omega_{s}\\ \dot{x}=\left[\begin{array}{c} {\dot{x}}_{o}\\ {\dot{x}}_{o}^{\prime }\\ {\dot{x}}_{t}\\ {\dot{x}}_{t}^{\prime } \end{array}\right]=\left[\begin{array}{c} x_{o}^{\prime }\\ \kappa_{v}\left(v-x_{o}\right)+\kappa_{t}\left(x_{t}-x_{o}\right)-\theta_{2}x_{o}^{\prime }\\ x_{t}^{\prime }\\ \frac{1}{4}\left(v-x_{t}\right)-\theta_{6}x_{t}^{\prime } \end{array}\right]+\omega_{x}\\ v=\exp\left(\theta_{7}\right)\cdot \cos\left(2\pi t+\exp\left(\theta_{8}\right)\right)+\omega_{v}\\ g\left(x_{t},x_{o}\right)=O\left(x_{t}\right)\cdot \exp\left(-{\left(\vec{r}+x_{o}-x_{t}\right)}^{2}\right) \end{array}$$

The generative model is very similar to Eq. (4), but with a few important differences. First, the subject's estimation of the target position *x*_*t*_ replaces its real world value **v**. Second, both the target and the centre of gaze are drawn (each with a degree of viscosity (*θ*_6_, *θ*_2_)) to the sinusoidally-varying (invisible) attracting location *v*, with parameters controlling its amplitude and phase lag (*θ*_7_, *θ*_8_). Third, there is no action; instead, eye movements are driven reflexively by descending predictions based upon the subject's beliefs that the centre of gaze is attracted to this invisible location, the target or both: *κ*_*v*_(*v* − *x*_*o*_) + *κ*_*t*_(*x*_*t*_ − *x*_*o*_). This means that the eye movements do not necessarily track the target itself but a point just ahead of the target — very much like focusing on the road ahead when driving (as opposed to the current location). Furthermore, the relative strengths of attraction to the invisible location and target are controlled by *κ*_*v*_ and *κ*_*t*_ respectively, whose values are partly dependent on whether the attracting location is behind the occluder (Fig. 1). This enables the model to make anticipatory eye movements; for example, if the target is occluded, the eye can track the invisible attracting location instead. The relative attraction of the invisible location and target – and the influence of the occluder – depends upon the (kinetic) parameters of each subject's generative model (*θ*_1_, *θ*_3_, *θ*_4_, *θ*_5_).

The resulting set up is shown on the upper right of Fig. 1: the generative model believes that the centre of gaze (blue circle) is attracted to the hidden location or cause (pink circle) and the target (red circle). The priors for the parameters are chosen such that when the occluder is present, the strength of attraction to the hidden location increases and the strength of attraction to the target decreases, as one might expect. Finally, the model parameters include the precision of random fluctuations at each level; namely, the sensory input (*ω*_*s*_), the motion of the hidden states (*ω*_*x*_) and the hidden cause (*ω*_*v*_). The prior expectations of the kinetic, precision and prior parameters were identical to those used in our previous study: see Table 1.

### Subjects

We acquired pursuit and MEG data synchronously from 17 healthy human subjects (mean age 25 years, age range 18–38 years, 10 female). All subjects were naïve to ocular pursuit tasks, had normal or corrected-to-normal vision, were right handed, had no history of neurological or psychiatric disorders and gave written informed consent. The study was approved by the UCL Ethics committee (1825/003). The experimental protocol was written in Matlab, using the Psychophysics and Eyelink Toolbox extensions (Brainard et al., 2006, Cornelissen et al., 2002) and Cogent 2000, developed by the Cogent 2000 team at the WTCN and ICN UCL, and Cogent Graphics developed by John Romaya.

### Stimuli and experimental paradigm

Each subject sat in an enclosed darkened magnetically shielded room, with their head partially stabilized within the MEG bore. The target was projected using a JVC DLA-SX21 projector (maximum refresh rate 60 Hz, resolution 1024 × 768 pixels) on to a screen of width 38.5 cm that was 57.5 cm from the subject. The target consisted of a black dot (2 mm across) surrounded by a white ring (3.5 mm radial width) moving over a black background (Fig. 1, top left). Total target diameter was 9 mm or 0.90° visual angle. Target luminance was 2.5 cd/m^2^ and background luminance was 0.01 cd/m^2^.

The target moved along a horizontal plane, halfway up the screen over 75% of the screen width (37.0° of visual angle). At the beginning of each trial, the target stimulus appeared at the left of its path, and remained stationary for 1–3 s (the precise time varied randomly). The target then moved horizontally, with a sinusoidal trajectory. One trial consisted of three full cycles of motion. In each trial, the target was occluded between the midline and the furthest 10% of the path from the starting position; i.e., for 40% (14.8°) of the total path. The occluder was the same colour as the background. The target had a period of 3.94 s and a maximum velocity of 20.3°/s.

The precision of the motion was varied between experimental conditions. In the ‘Smooth’ motion condition, the target moved sinusoidally. In the ‘Noisy’ motion condition, a Gaussian random walk of variance *σ*^2^ = exp(− 0.5) was added to the phase of the target motion, such that:

6 $$\begin{array}{c} x\left(t\right)=\cos\left(2\pi f\left(t+\phi \left(t\right)\right)\right)\\ \phi \left(t\right)=\phi \left(t-1\right)+\omega \left(t\right)\\ \omega \left(t\right)\sim N\left(0,\sigma^{2}\right) \end{array}$$

Here *f* is the target frequency and *t* the time in milliseconds. This created rapid random fluctuations around an underlying sinusoidal motion that had approximately the same period as the Smooth trajectory. The ensuing fluctuations were too fast to be tracked with the eyes, and subjects were instructed to follow the ‘average’ position of the target, rather than the fluctuations themselves. Subjects were explicitly asked to maintain pursuit and not to saccade to the side of the occluder. The experiment consisted of 10 blocks of 8 trials, such that there were 40 trials (120 cycles) of each of the two conditions. Smooth and Noisy stimuli were presented in pseudorandom order, such that every eight trials contained four of each.

### Eye movement data recording and analysis

Eye movement data – including horizontal and vertical eye movements and pupil diameter – were collected using an infrared eyetracker (Eyelink 1000, SR Research, Ontario, Canada), sampling at 1000 Hz. The eyetracker was recalibrated using an automated calibration routine after every block of 8 trials; this entailed the presentation of a 5 mm white circular target stimulus of luminance 0.5 cd/m^2^ at ± 16° horizontal, ± 10° vertical and 0° of visual angle, until the calibration error was < 1°. The pursuit trajectory root mean square errors were calculated for each cycle, and those over 5.6 cm were visually inspected. If there was evidence of either a calibration problem or gross distortion from blinking (or complete failure to track the target) the cycle was discarded (< 10% total cycles were discarded for any subject). For subsequent modelling, the remaining pursuit trajectories – containing both smooth pursuit and saccades – were averaged over cycles and subjects to create grand averaged responses. The grand averages were normalised so that they corresponded to a single cycle of target motion with unit amplitude. Grand averaged plots of smooth pursuit eye movement (SPEM) velocity (i.e. excluding saccades) were generated by removing all eye movements over ± 35°/s from eye velocity plots and averaging the remainder over cycles and subjects (note that these velocity plots are for display purposes only: model fitting used the pursuit trajectories with smooth pursuit and saccades).

### MEG data recording and preprocessing

MEG data were recorded continuously from 274 axial gradiometers using a CTF Omega whole head system at a sampling rate of 600 Hz. Head position was measured using three localization coils, one at the nasion and two pre-auricular bilaterally, at the beginning of each block. Stimulus onset was timed as the emergence of the target from the occluder at the centre of the screen (i.e. moving leftward). The MEG data were processed using SPM12b (http://www.fil.ion.ucl.ac.uk/spm/software/spm12, Litvak et al., 2011); bandpass filtered at 0.5 to 49 Hz, and epoched from − 300 ms pre-stimulus to 500 ms post-stimulus onset. The epochs were mean corrected over the whole epoch, and visually inspected for artefacts, which were rejected using FieldTrip ‘rejectvisual’ (http://www.fieldtrip.nl, Oostenveld et al., 2011). Artefact rejection was performed blind to the experimental condition.

### MEG data eye movement artefact removal and averaging

**Fig. 2:**
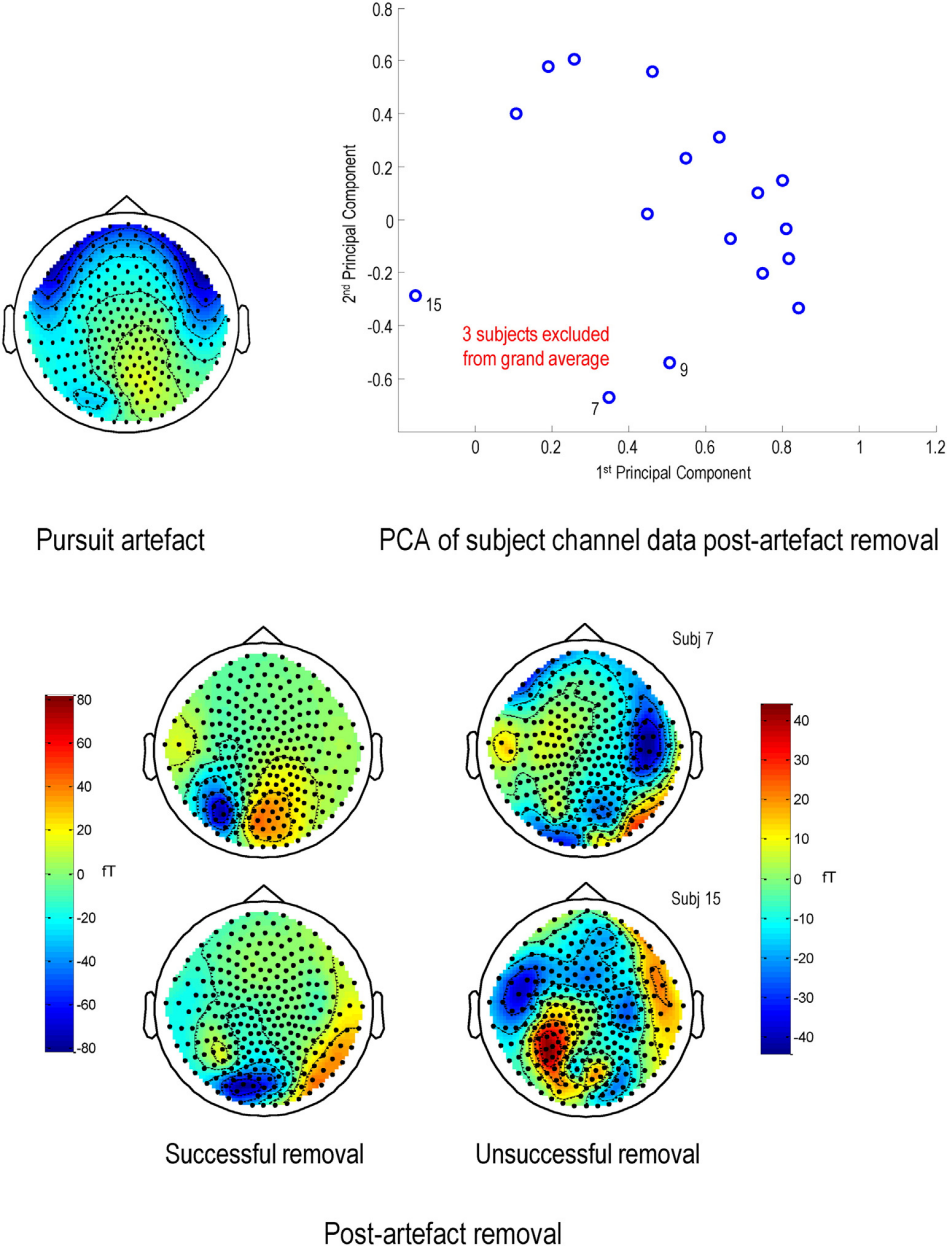
MEG data pursuit artefact removal and subject exclusion. The topoplot on the top left is an average over 0–200 ms of the first eight subjects' MEG data and both conditions. It shows the extent of the pursuit artefact (the inverted U-shaped blue band across the anterior regions). The four topoplots at the bottom show individual subject data, averaged over 0–200 ms of the Smooth condition, after pursuit artefact removal (units are in femtoTesla). Pursuit artefact removal seemed successful on inspection of most subjects' topoplots: two examples are the pair on the left. However, three subjects still appeared to be compounded by pursuit artefacts — two are shown on the right. The graph on the top right plots the first two principal components of individual subjects' MEG data, averaged over conditions. The three subjects on the bottom left were subsequently treated as outliers and excluded from the MEG grand average.

**Fig. 3:**
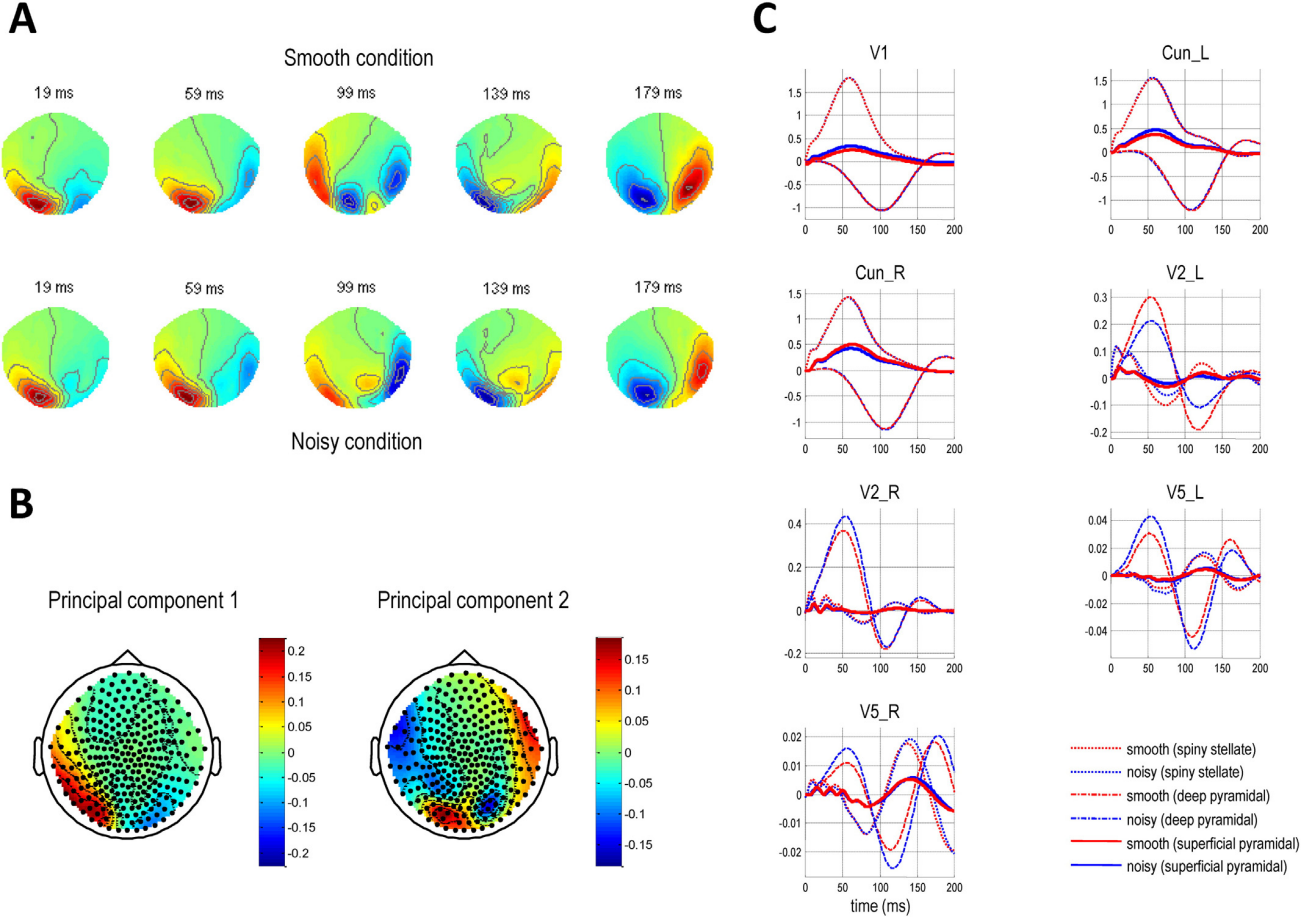
Evolution and principal components of the evoked fields. A: The evoked response fields, averaged across all subjects, are plotted here at various time points in the 0–200 ms time window. The Smooth condition responses are on the top row, and the Noisy condition responses on the bottom. B: The first two principal components of the data matrix **M** – i.e. the first two columns of **U** – are plotted on two topoplots. The second principal component contains substantial eye artefact signal. C: The evoked responses at source level, for the grand averaged data. The neuronal populations whose activity is plotted on each graph (the spiny stellate cells and superficial and deep pyramidal cells) are those contributing to the MEG signal (i.e. omitting the inhibitory interneurons — see also Fig. 4).

Visual artefact rejection eliminated trials containing blinks and other gross sources of signal distortion, but all remaining trials had significant pursuit artefacts (Fig. 2, top left, illustrates this artefact on a topoplot of the averaged responses of the first eight subjects). To estimate the spatial signature of the pursuit artefact, we performed a mass univariate regression of all MEG channels on eye position in the averaged ERF. These components were then removed using signal space projection (Uusitalo and Ilmoniemi, 1997) in ‘spm_eeg_correct_sensor_data’, using the standardised regression coefficients (weighted by the standard deviation; Bauer et al., 2010). This method was successful in most cases (Fig. 2, bottom panel, left hand topoplots). However, a few datasets still contained some artefact (Fig. 2, bottom panel, right hand topoplots); please see below. After artefact suppression, the data were merged and baseline corrected for the pre-stimulus period (− 300 to 0 ms). Trials of each condition (Smooth versus Noisy motion) were then averaged within each subject and then over all subjects to form a grand average (Fig. 3A), as with the behavioural (eye tracking) data.

### MEG source localisation

The cortical areas involved in smooth pursuit are well known in humans (Lencer and Trillenberg, 2008), and include early visual areas (e.g. V1 and V2), motion-sensitive V5, the frontal eye fields (FEF) and the parietal eye fields (PEF).

To define precise prior locations for subsequent DCM analysis, multiple sparse priors were used to estimate the cortical sources of the ERF (Friston et al., 2008). A tessellated cortical mesh template surface – in canonical (MNI) anatomical space – served as a brain model to estimate the current source distribution (Mattout et al., 2007). This dipole mesh was used to calculate the forward solution using a single shell head model. The inverse solution was computed for two grand averaged conditions separately, although they revealed the same sources. Their peak MNI coordinates were obtained from viewing the reconstructed sources on a canonical template in SPM12, and the corresponding brain areas were identified using the Anatomy toolbox for SPM (Eickhoff et al., 2005). The areas included: central V1 and bilateral cuneus (peripheral V1) and V2 (see the first five rows of Table 2 for their MNI coordinates). We also obtained sources which corresponded to bilateral V5, but whose inferred location had shifted anteriorly (to − 50 − 49 10 (V5 L) and 46 − 49 10 (V5 R)), presumably due to residual pursuit artefact distortion. When five subjects with probable pursuit artefact on their topoplots were excluded from the grand average, these source locations moved to bilateral V5 (− 40 − 72 − 3 (V5 L) and 40 − 67 0 (V5 R)).

**Fig. S1:**
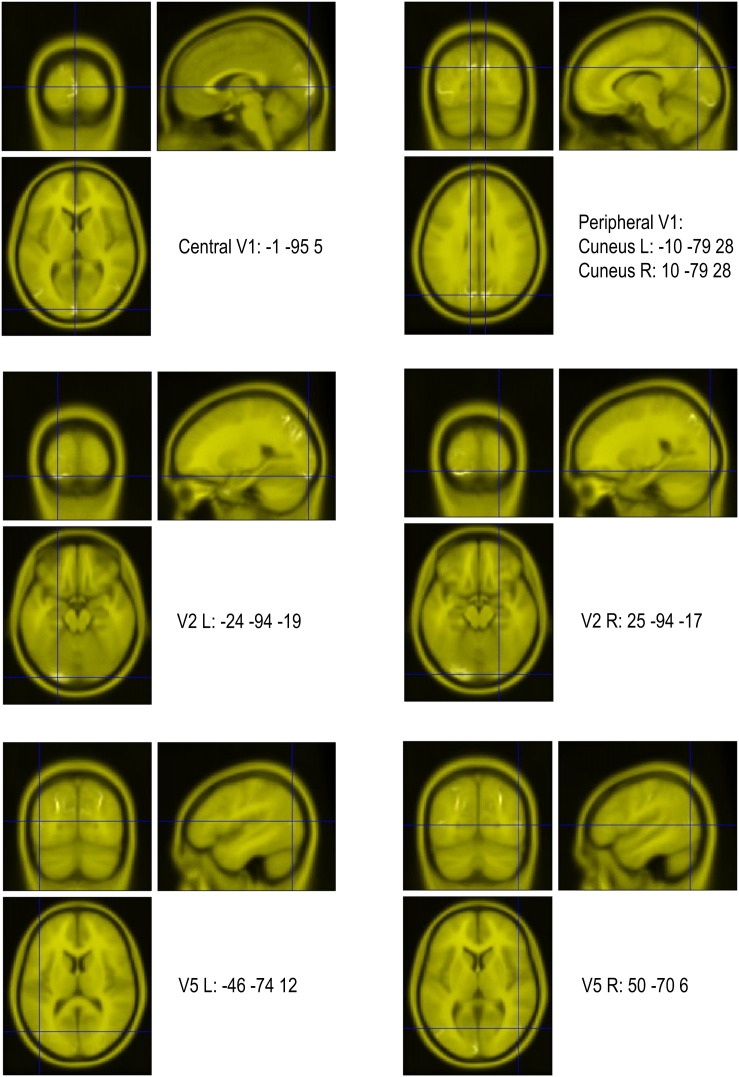
Source localisation results. The sources used for subsequent dynamic causal modelling analysis are shown here as the peak activations with their MNI coordinates. They are overlaid on a canonical T1 weighted brain image.

To segregate subjects with pursuit artefacts in a more principled way, we used Principal Component Analysis (PCA) to distinguish between subject groups with similar activity patterns (i.e. eye artefacts) in their topoplots. We averaged each subject's data over all time points and conditions and the resulting vectors were normalised to unit sum of squares and concatenated into a mean-corrected data matrix **M** of 274 channels × 17 subjects. A singular value decomposition **M** = **UΣV**^*T*^ was performed and a projection of the data **P** = **M**^*T*^**U** onto the first two principal axes is shown in Fig. 2 (upper right). The PCA segregates the subjects with eye artefacts, which contribute most to the second principal component (Fig. 3B), and so the three (outlier) subjects on the bottom left (Fig. 2) were excluded from the MEG data grand averages. When source localisation was repeated on the remaining 14 subjects, sources were again found in bilateral V5 (bottom two rows of Table 2). Some parietal sources were also apparent in this localisation (visible in Inline Supplementary Fig. S1, bottom panels) but we did not want to overcomplicate our DCM by adding them, as our hypothesis centred on precision encoding in early visual cortex. FEF did not appear in either source reconstruction.

Inline Supplementary Figure S1

### Model comparison with DCM

Dynamic causal modelling is a Bayesian model inversion and selection scheme that uses standard Bayesian (variational) procedures to estimate the parameters of time series models (Friston et al., 2003). In DCM for ERFs, the model is specified in terms of differential equations describing the dynamic responses of a network of coupled sources to sensory input, where experimental manipulations can change certain connectivity parameters within or between sources (David et al., 2006). The intrinsic dynamics of the sources themselves are based on neural mass models of neuronal subpopulations.

**Fig. 4:**
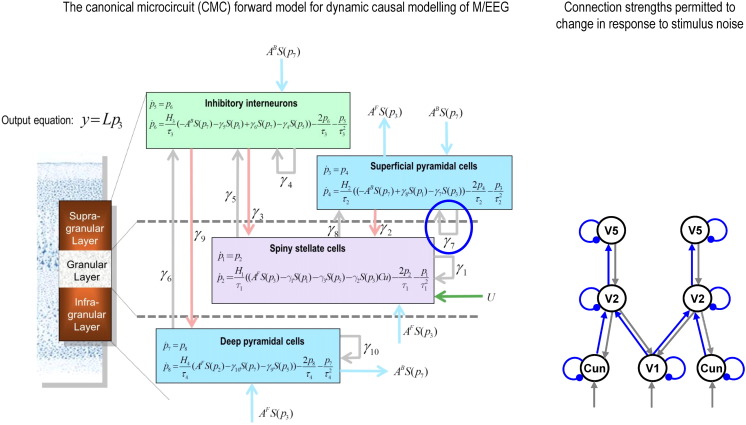
Canonical microcircuit (CMC) model for DCM and connections modulated by stimulus noise. The left panel illustrates the forward model of how cell populations interact both within and between cortical areas, and how their changing conductances generate the MEG signal. DCM inverts this model and estimates the connectivity parameters describing the strength of forward and backward connections (light blue arrows) and intrinsic or self-inhibitory connections (circled in blue), which control the excitability or gain of the superficial pyramidal cell population. The sensory input, if present in a given area, enters the spiny stellate population, here illustrated with a green arrow. The CMC model improves upon past forward models that only contain three populations – i.e. those that group superficial and deep pyramidal cells together – because superficial and deep layers are the sources of forward and backward connections (i.e. of prediction errors and predictions, in predictive coding) respectively (Bastos et al., 2012). Reproduced from a personal communication from Dr Harriet Brown. The right panel shows the DCM structure, with the (forward and intrinsic) connections that could be modulated by noisy target motion highlighted in blue. Sensory inputs enter all three sensory sources. Please note that lateral connections between identical areas were included at all levels (including between central V1 and bilateral cuneus; i.e., peripheral V1), but are not shown for clarity. In DCM, extrinsic forward, backward and lateral connections are excitatory, while self-connections are inhibitory. Excitatory connections end in arrows, and inhibitory connections in balls. Cun — cuneus, i.e. part of V1 representing the peripheral visual field.

We used the canonical microcircuit (CMC) model (introduced by Bastos et al. (2012) and Bastos et al. (2015) and applied by Boly et al. (2011) and Moran et al. (2013)), in which superficial and deep pyramidal cells are parameterised separately (along with stellate cells and inhibitory interneurons — see Fig. 4): this is important as these populations are the sources of forward and backward connections respectively. The purpose of the CMC model is to model cortical activity using a (minimal) network that could implement predictive coding; in that it can encode prediction errors and predictions and pass these messages forwards and backwards (respectively). The CMC model is based as much as possible on established interlaminar connection probabilities (Haeusler and Maass, 2007, Thomson et al., 2002); although in order to allow it to function as a predictive coding circuit, some adaptations had to be made, detailed in Bastos et al. (2015). For example, the CMC model confines forward connection input to spiny stellate cells (it does not model the small input to infragranular layers, or thalamic input to layer VI), and condenses some excitatory and inhibitory neurons into single (respective) populations.

Hierarchical arrangements are important in DCM for M/EEG because the equations describing forward and backward connections have different effects on their target populations: forward connections (ostensibly conveying prediction errors) are driving and linear, whereas backward connections (conveying predictions) are both driving and modulatory (Bastos et al., 2012, David et al., 2005, Jansen and Rit, 1995). The sensitivity of each population to its afferents is controlled by a self-inhibition parameter (one is circled in Fig. 4), which combines the effects of voltage-gated potassium currents, calcium-gated potassium channels and recovery from fast sodium current inactivation to reduce postsynaptic gain. These and other parameters of the model can be estimated from MEG data using a forward model that maps source activity to sensors (Kiebel et al., 2006), given priors over source locations. Source locations are themselves optimised during model inversion. For computational efficiency, the sensor data was reduced to the eight principal modes of the prior covariance before fitting DCMs to channel data between 0 ms and 200 ms post-stimulus time (Fig. 3C). Dynamic causal modelling furnishes estimates of model parameters and variational free energy, which approximates the log model evidence. This model evidence comprises both accuracy and complexity. Therefore, models with the greatest evidence provide the simplest and most accurate explanation for observed data (i.e. do not overfit).

### DCM analysis of the effect of target motion noise

The MEG source localisation procedure is described in Methods Section 2.8. Having established these sources, we then sought to determine the changes in intrinsic and extrinsic connectivity induced by imprecise target motion. To organise our model space we used a 2x2x2x2 factorial design looking at two types of connections – forward and intrinsic – in the upper (V5) and lower (V1/V2) levels of the visual hierarchy (Fig. 4, right panel). This model space reflects our previous finding that noisy target motion should increase lower level or sensory precision (Adams et al., 2015). This increase in sensory precision has been associated with a reduction in self-inhibition of superficial pyramidal cells in DCMs of cortical responses (Brown and Friston, 2012, Brown and Friston, 2013, Moran et al., 2013). One could also hypothesize that noisy motion would decrease higher level precision or increase the influence of ascending prediction error; i.e., the strength of forward connections. We had no hypothesis about lateral connections, and backward connections play a dominant role in cortical responses from around 300 ms post-stimulus onset but not as early as 200 ms (Garrido et al., 2007). To assess the effect of noisy target motion, we compared DCMs that allowed for specific changes in connectivity using the grand averaged data (excluding the three outlier subjects — see Section 2.8).

### Dynamic causal modelling of oculomotor pursuit

The pursuit DCM works in the same way as DCM for imaging data, in that it uses a generative model of (Bayes-optimal) behavioural responses, as opposed to neuronal responses. The inversion scheme used in this application is also the same as the scheme used to invert imaging data timeseries: see previous studies (Friston et al., 2003, Friston et al., 2007, Kiebel et al., 2009) for details. The behavioural DCM predicts the position error (i.e. the difference between the angular directions of gaze **x**_*o*_ and target **v**: Fig. 1) $a=x_{o}\left(\tilde{\mu }\right)-v+e$, given some generative model parameters. Note that the position error (a sensory quantity) is described as action *a* because under active inference, action just enacts sensory predictions (by minimising sensory prediction errors) — either through classical reflexes or a learned inverse model (as here). Thus we obtain the likelihood of the observed position error (averaged over multiple trials), under the assumption of additive Gaussian noise *e* ~ *N*(0, *Σ*(*θ*_*o*_)):

7 $$\begin{array}{c} p\left(a|\eta ,\theta_{o},m_{0}\right)=N\left(a^{*},\Sigma \left(\theta_{o}\right)\right)\\ a^{*}=x_{o}\left(\tilde{\mu }\right)-v \end{array}$$

The first equality says that the likelihood of the position error depends on both the Bayes-optimal position error predicted by the subjective model and the observation noise. Prior beliefs about the parameters of the observation model then provide a full generative model of observed behaviour, which can then be inverted (see Eq. (2)). Table 1 contains the prior expectations, while the prior variance of the (log scaling of the) parameters was set to one half, rendering the priors relatively uninformative. Model inversion provides the log model evidence *F*_*o*_ ≈ − ln *p*(*a*|**v**, *m*_*o*_), which is then used in Bayesian model averaging, to weight the value of a particular model's parameters by the likelihood of that model. We can therefore characterise the effects of experimental manipulations on parameters using the Bayesian model averages over all possible models. The procedure is described in more detail in Adams et al. (2015).

## Results

### Behavioural DCM

**Fig. 5:**
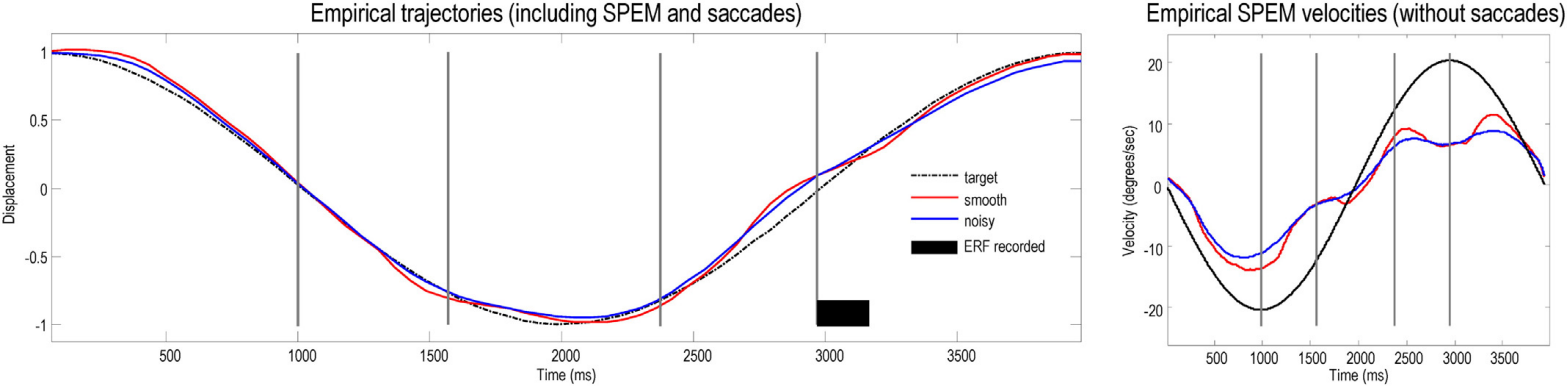
Grand averaged plots of eye trajectories and SPEM velocities. The left graph shows the grand averaged eye displacement for the two experimental conditions, Smooth (red) and Noisy (blue) target motion; the target path is shown as a dotted black line. The presence of the occluder is indicated by the vertical grey lines. When the target emerges from its second pass through the occluder, the ERF is recorded (black bar). The amplitudes of the traces have been normalized to ± 1. The right graph shows the grand averaged SPEM velocities, with saccades (defined as movements faster than ± 35°/sec) removed from the velocity data prior to averaging.

**Fig. 6:**
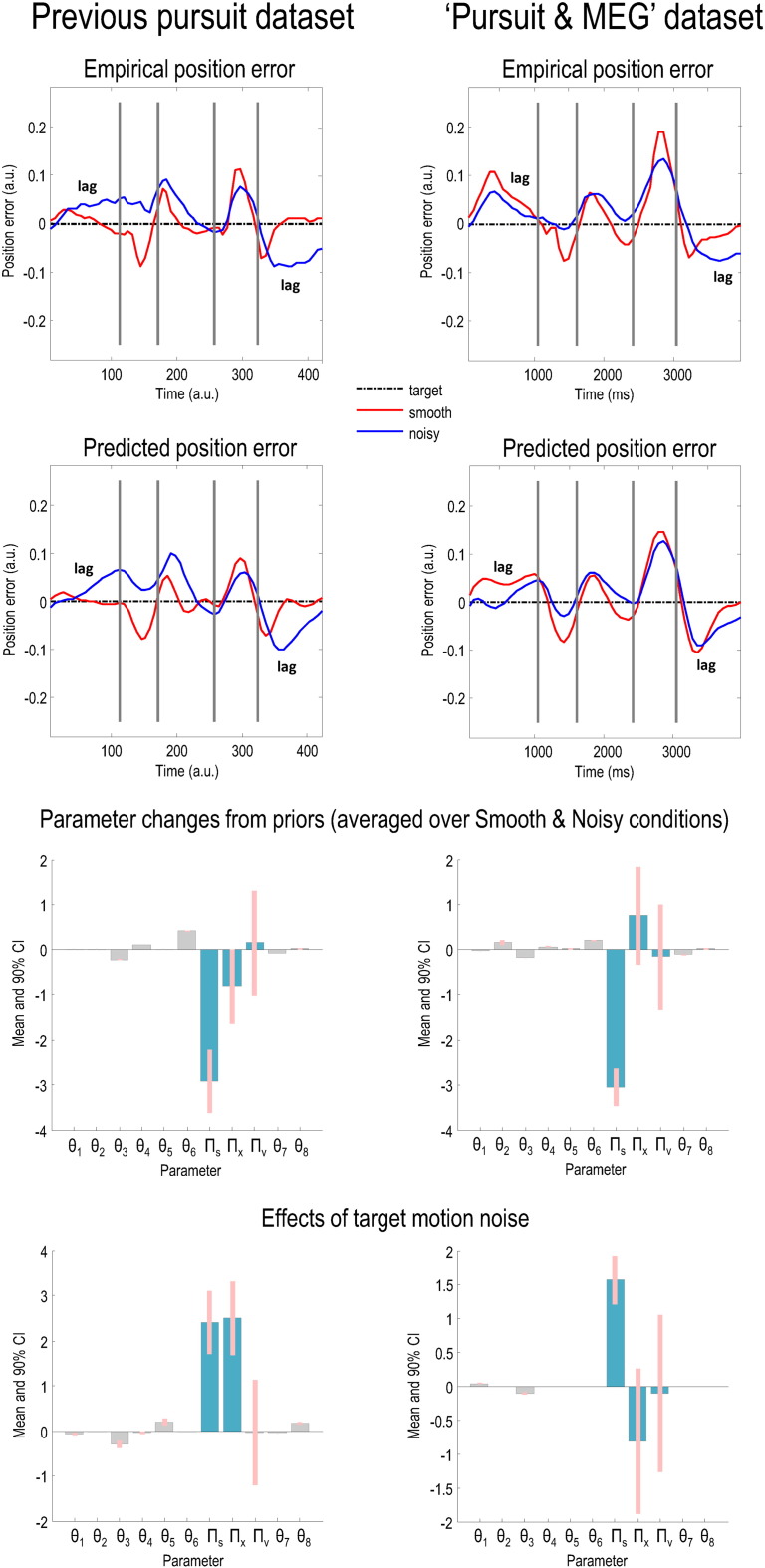
Comparison of empirical and predicted position errors and parameter estimates in this and a previous dataset. The graphs on the first row show empirically observed position error (target position — eye position) in arbitrary units (the traces have been normalised with respect to displacement) for both Smooth (red line) and Noisy (blue line) conditions. Note that being behind the target entails being above the black line in the first half of the cycle and below it in the second. It is clear that the pattern of eye movements in each condition is very similar in both experiments; the major difference is an increase in lag in the Smooth condition in the second experiment, especially in the first quarter cycle (please see the main text for discussion of this phenomenon). The graphs on the second row show the position errors predicted by the generative model in Fig. 1, using the posterior expectations of the parameters in the lower two rows: in both experiments, the models fit the data well. The previous experiment (left panels) used two different speeds and hence the plots on the left have been normalised with respect to time, but those on the right – using only one speed – have not. The graphs on the third and fourth rows depict the parameters used to generate the predicted position errors on the second row. The graphs on the third row display the posterior expectations of the model parameters (averaged over conditions), plotted as the changes from prior expectations listed in Table 1. The graphs on the fourth row display the changes in parameters due to the noise of target motion. The changes in kinetic parameters (*θ*_1_, …, *θ*_6_) are absolute, but the changes in precision parameters (teal) and prior parameters are log scaled. The pink bars correspond to 90% Bayesian confidence intervals. The posterior expectations in each dataset are remarkably similar: please see the text for a discussion of their minor differences.

The empirical eye trajectories in Smooth and Noisy conditions are plotted on the left of Fig. 5 and the eye velocities (excluding saccades) are shown on the right. When the target was occluded, smooth pursuit eye movement (SPEM) velocity in both conditions decreased to a steady ‘residual predictive velocity’ of either − 3°/s (decelerating target) or 6°/s (accelerating target). The corresponding position errors between eye and target in each condition are shown on the top right of Fig. 6, together with the predictions of the pursuit model below (second row, right column). For comparison, the results of an earlier behavioural experiment (Adams et al., 2015) conducted with the same stimuli are displayed on the top left of Fig. 6, with the model predictions following DCM inversion below it. The model predictions of these independent data are reasonably accurate in both cases and consistent with each other (see the Discussion for comments on the few inconsistencies).

Precision parameters and prior parameters (*θ*_7_, *θ*_8_; governing the amplitude and phase lag of the target) were estimated in terms of their log scaling — such that a value of 0 corresponds to a scaling by exp(0) = 1 or no change from the prior expectations in Table 1. Kinetic parameters (*θ*_1_, …, *θ*_6_**;** governing how much the eye is attracted to either the target or the location ahead of the target, and the eye's viscosity, i.e. velocity-dependent forces — as opposed to any viscous properties of the eye) were estimated in terms of their absolute change. In addition to estimating all parameters (averaged over conditions), we also estimated the changes induced by target motion noise. The changes in the parameter estimates from their prior expectations are displayed in the third row (right column) of Fig. 6, and the effect of target noise is shown on the bottom right. For comparison, the results of our previous experiment are provided in the third and fourth rows of the left column.

The key points to take from Fig. 6 are: i) except for a reduction in sensory precision, there were minimal changes in the posterior expectations of the parameters (averaged across conditions) from their prior values, just as in our previous experiment (third row); ii) target motion noise had minimal effects on the model parameters except for sensory precision ln *Π*_*s*_, which changed between conditions by a factor of exp(1.57)^2^ = 23. The only difference between these results and our previous findings was that ln *Π*_*x*_ also changed in the latter (fourth row) — in this experiment its 95% confidence intervals overlapped zero.

### Biophysical DCM results: modulation of connectivity by noisy target motion

**Fig. 7:**
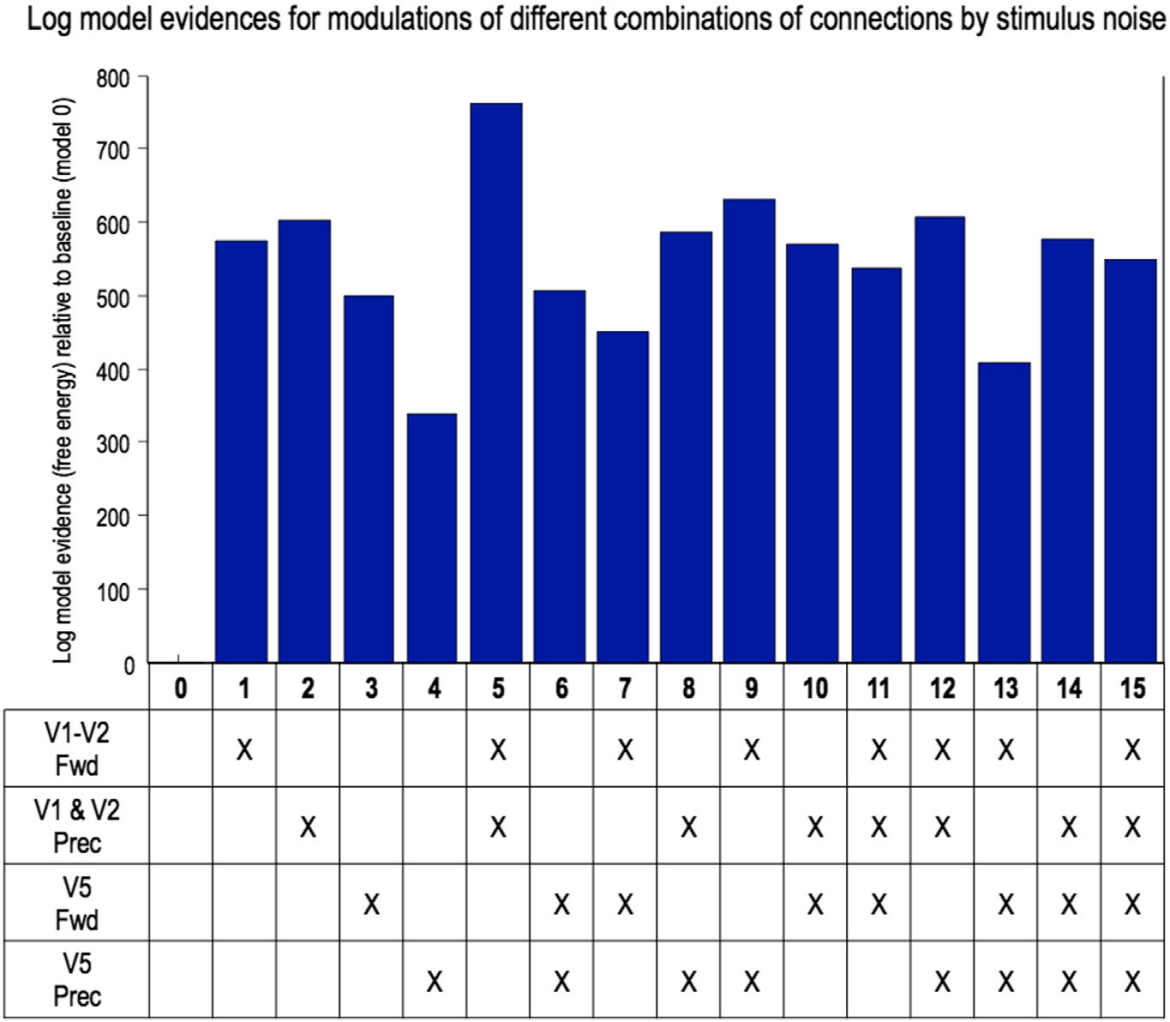
DCM results. This graph plots the log model evidences for each combination of two factors: connection type (precision, i.e. self-inhibitory, and forward) and hierarchical level (low, i.e. V1/2 and high, i.e. V5). Model 5 — in which only lower connections (both forward and self-inhibitory) are modulated by stimulus noise — is the clear winner, with > 100 times the evidence of the runner up.

The results of the DCM analysis of connections that are most likely to be modulated by noisy visual motion are shown in Fig. 7. The winning model is Model 5, which allowed for changes in lower level recurrent self-inhibitory (precision) and forward connections. The evidence for Model 9, which allowed changes in lower level forward and higher level self-inhibitory connections, was 135 times less, which reflects ‘decisive’ evidence for Model 5.

**Fig. 8:**
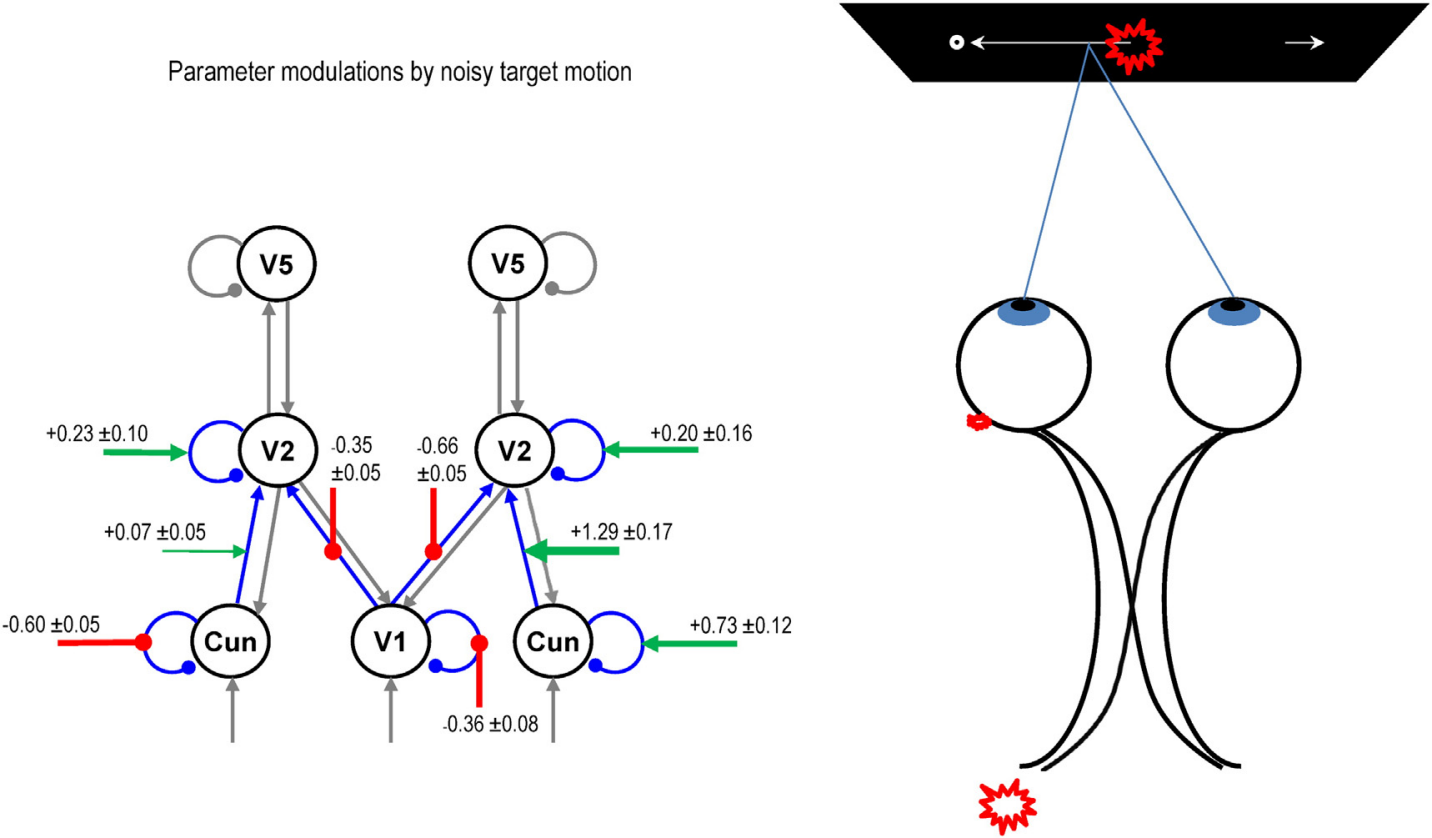
Modelling grand averaged data with the winning model. The left panel displays the posterior expectations of the modulation (log scaling) of the self- and forward connectivity by noisy target motion in the winning DCM (Fig. 7) of the grand averaged MEG data. On average, noisy motion induces a disinhibition of central V1 and left cuneus (peripheral V1), but increased self inhibition of right cuneus. In addition, noisy motion diminishes forward connectivity from central V1 but increases it from peripheral V1. Grey and blue arrows denote excitatory connections, balls denote inhibitory connections. Cun — cuneus (peripheral V1). The right panel illustrates a possible reason for the laterality of the V1 gain (self-inhibition) changes: because on average subjects' eyes are ahead of the target (in both Smooth and Noisy conditions) when it emerges from the occluder (around 3000 ms on Fig. 5, left panel), its image is concentrated in the right hemifield and hence left visual cortex.

The DCM results of Model 5 are shown in Fig. 8 (left panel). The logarithms of the changes in connectivity due to stimulus noise are shown on the red and green arrows. There is a robust disinhibition of self-connections in central V1 and left cuneus, and a similarly substantial increase in self-inhibition in right cuneus. There are increases in self-inhibition in bilateral V2. The strength of forward connections from central V1 to bilateral V2 is decreased, but from right cuneus to right V2 there is a large increase.

### Correlations between behavioural and biophysical DCM results

**Fig. 9:**
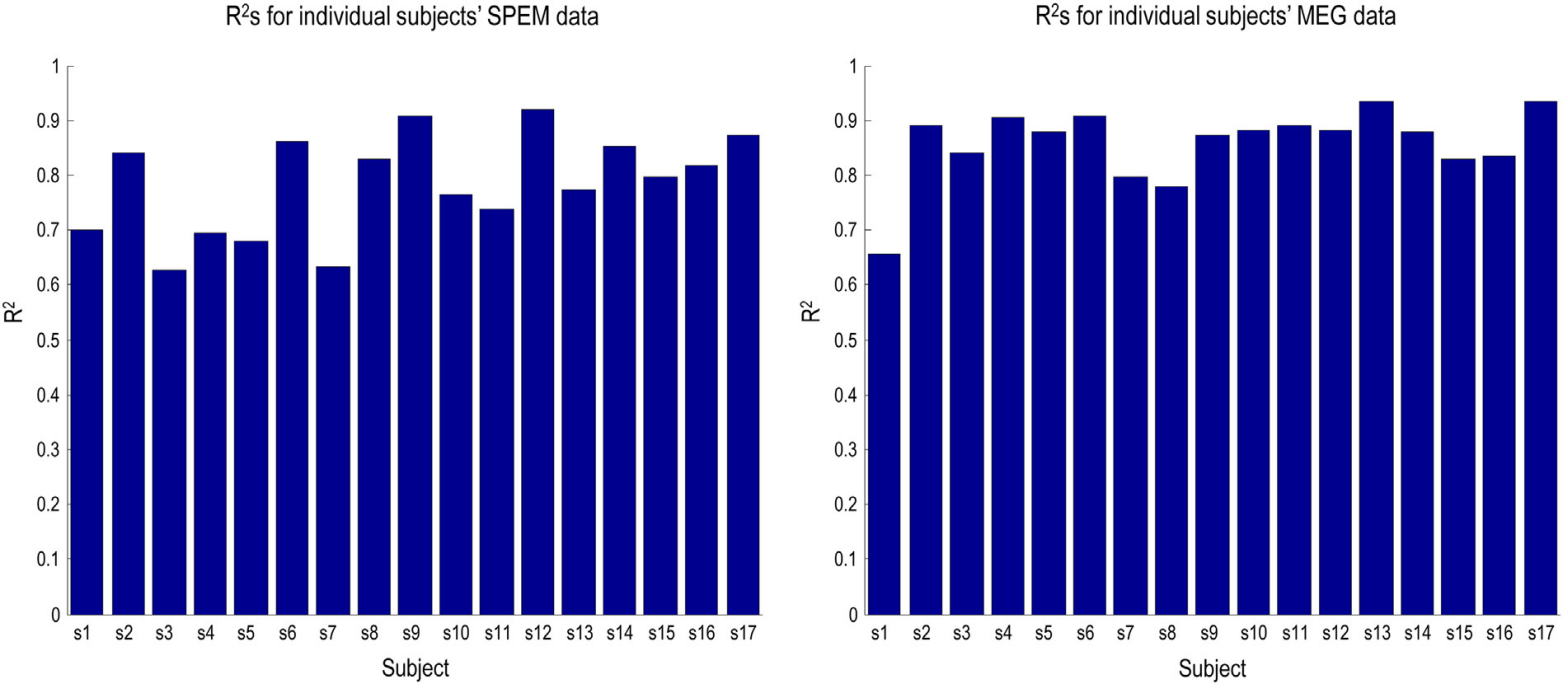
R^2^ values for modelling individual subjects' data. The bar charts display R^2^ (coefficient of determination) values for modelling individual subjects' data: on the left, with pursuit DCMs, on the right, with MEG DCMs (using Model 5).

Model 5 was fitted to individual subjects' MEG data for the Smooth and Noisy conditions using the values in Table 2 as priors on source locations, which were optimised during model inversion. For this analysis we included the three subjects we previously excluded from the grand average, as our previous concern that eye movement-related artefact might distort signal around V5 (and e.g. alter our priors specified for source locations) was less relevant to investigating the effect of precision in V1. The pursuit trajectory averages for each condition in individual subjects were fitted to behavioural DCMs using the same priors over model parameters in Table 1. The R^2^ (coefficient of determination) values for each subject's behavioural and biophysical DCM fits are shown in Fig. 9. The pursuit DCM model fits vary in their quality from reasonable (R^2^ = 0.6) to very good (R^2^ = 0.9), the average being 0.78. The biophysical MEG DCM model fits are even better, with an average of 0.86.

**Fig. 10:**
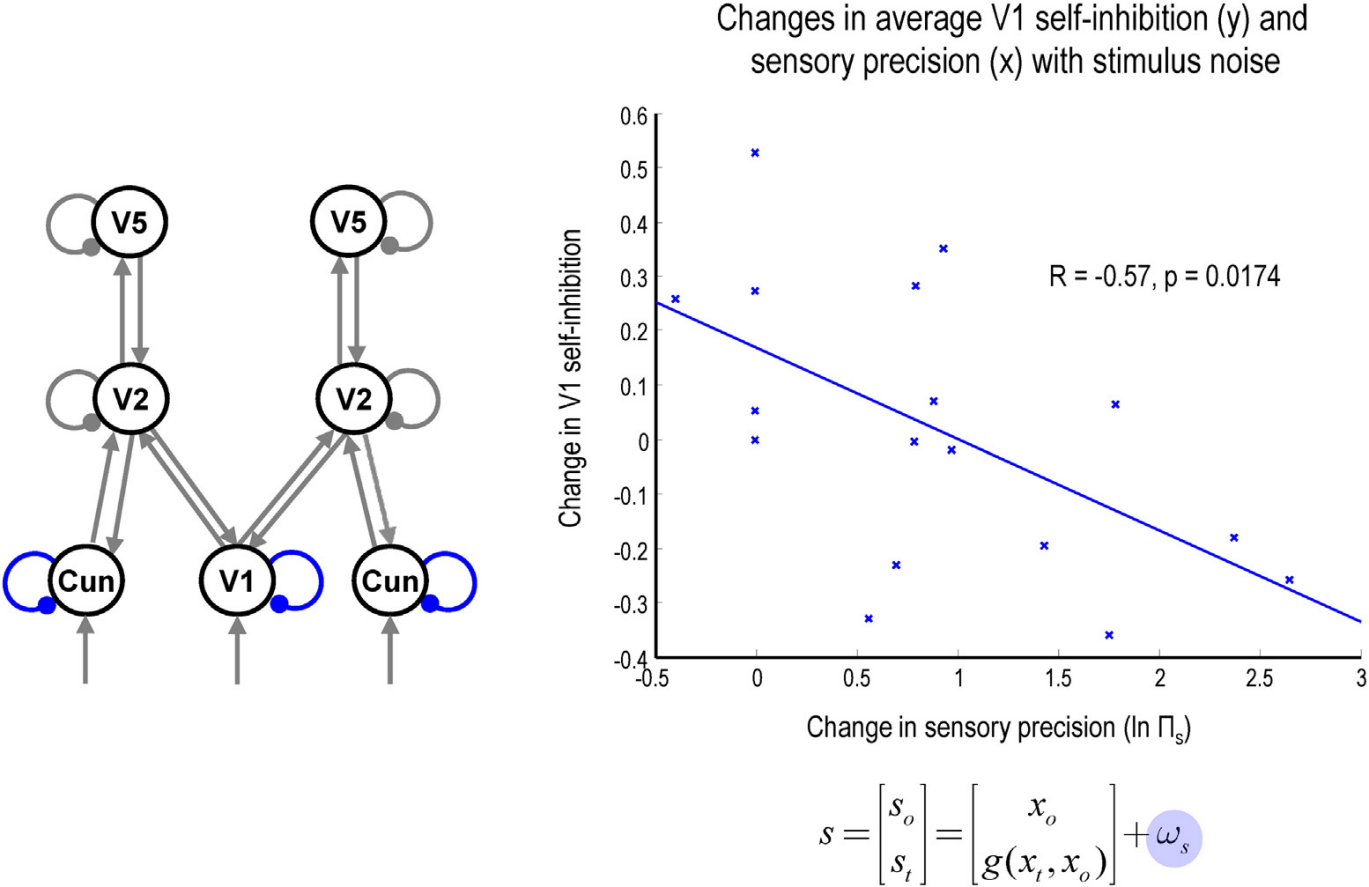
Correlation of the two precision metrics in individual subjects. The graph (centre) shows the correlation between noise-induced changes in sensory precision – estimated by the pursuit DCM – and mean changes in the gain (self-inhibition) of pyramidal cells in V1 (left), estimated by the MEG DCM. Sensory log-precision (ln *Π*_*s*_) is the subjective expectation of inverse variance of random fluctuations at the sensory level (*ω*_*s*_, highlighted below). The full model is described in Fig. 1.

The correlations between the noise-induced changes in sensory precision ln *Π*_*s*_ in the pursuit DCM and changes in mean V1 or V2 self-inhibition in the MEG DCM were evaluated. We hypothesized a correlation between sensory precision and self-inhibitory connectivity (rather than forward connectivity) because precision is usually associated with superficial pyramidal cell gain (i.e. self-inhibition) in the dynamic causal modelling of cortical responses (Brown and Friston, 2012, Brown and Friston, 2013, Moran et al., 2013). There was a negative correlation (R = − 0.57, p = 0.0174) between changes in sensory precision and mean V1 self-inhibition (Fig. 10). This correlation remains significant after Bonferroni correction for multiple comparisons.

This is a pleasing result because these independent measures of (putative) precision or gain were based upon completely independent data and, furthermore, data that were fundamentally different in nature (eye tracking and MEG data). The strength of this correlation speaks not only to the validity of precision as a quantity that bridges between the computational and physiological explanations for subject responses — but also speaks to the validity of the generative model-based methods used to estimate precision in V1. There was no correlation between sensory precision and mean V2 self-inhibition (R = 0.35, p = 0.169).

In a post-hoc analysis, prompted by the asymmetrical pattern of results in Fig. 8 (left panel), we found the relationship between changes in sensory precision and mean V1 self-inhibition was driven by the negative correlation between sensory precision and left cuneus (peripheral V1) self-inhibition (R = − 0.61, p = 0.0096). There were no significant correlations between sensory precision and self-inhibition in central V1 (R = − 0.15, p = 0.56) or in right cuneus (R = − 0.02, p = 0.95).

It is possible that the correlation between sensory precision and left cuneus (V1L) self-inhibition was confounded by the effect of noise on eye position error. To exclude this possibility, we included the position error of the eye as an additional regressor in a linear (regression) model of the effects of target noise. The only significant predictor of the effect of target noise on V1L self-inhibition was sensory precision: *β* = − 0.40 (SE = 0.13), *t*(14) = − 2.98, p = 0.01, F-statistic vs constant model = 4.52, p = 0.03.

## Discussion

We have shown that oculomotor tracking of a visual target with imprecise motion leads to eye movements that can be explained by increased sensory precision within a hierarchical predictive coding model of pursuit eye movements. Most importantly, the sensory precision estimated from the eye movements correlates, across subjects, with its supposed neurobiological substrate; namely, the synaptic gain in V1 superficial pyramidal cells (estimated using biophysical modelling of visual evoked response fields). This is the first use of DCM to invert models of behaviour and imaging data obtained concurrently, and is a prequel to developing fully integrated models of behaviour and brain responses. We now discuss our results in more detail in terms of the four predictions (two replications and two hypotheses) in the introduction:

Our first replication concerned the behavioural consequences of our experimental manipulation; namely, that noisy target motion would increase the lag, when the target is visible, and reduce anticipatory saccadic movements, when it is occluded.

Target noise did increase the lag of the eye behind the target in the last quarter of the cycle, and also diminished the size of the anticipatory saccadic movements during target occlusion, as we have previously reported (Fig. 6, first row). The main difference between this and the previous experiment was in the first quarter of the cycle — when the eye lags the Smooth more than the Noisy target. This is unusual, as subjects normally track the Smooth target perfectly when it is visible. The reason for this difference is unclear: inspecting the raw data, the lag at the 300 ms time point increases as the experiment goes on, so there is an effect of fatigue (the MEG study required many more trials than the behavioural study), but this does not apply disproportionately to Smooth trials (correlation between lag and cycle number, r = 0.29, p = 0.04) over Noisy trials (r = 0.32, p = 0.07).

The effect of target noise on SPEM velocity (ignoring saccades) was more definitive: throughout the cycle, in the Noisy condition, SPEM velocity was lower than in the Smooth condition — whenever the target was visible and moving at greater than ± 8°/s. As we found previously, target noise does not affect residual predictive velocity (i.e., when the target is occluded).

Our second replication was that target motion noise would increase sensory precision, as inferred from eye trajectories by our pursuit DCM. The pursuit DCM parameter estimates for this and our previous study (Fig. 6, third and fourth rows) are very similar. As previously, the average effect of watching a sinusoidal target is to lower sensory precision relative to prior precision (third row, right column) — because the target trajectory is predictable. However, this effect is reversed with noisy target motion (fourth row, right column).

**Fig. S2:**
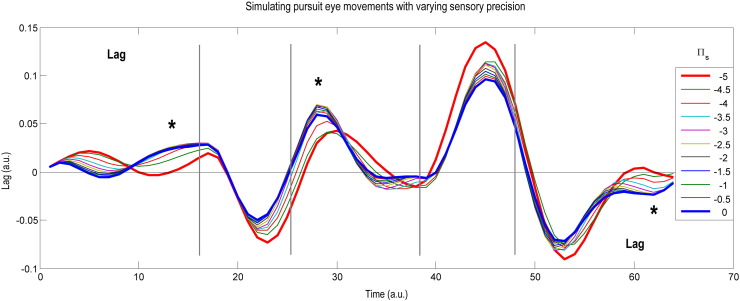
Simulating pursuit movements with varying sensory precision. This figure illustrates some simulated pursuit movements using the same format as Fig. 5: i.e. the y axis plots the lag (target position (the flat black line) — eye position), rather than its absolute displacement. For these simulations, all parameters except the log precision at the sensory level (ln *Π*_*s*_) were kept constant at the posterior values plotted in the upper left panel of Fig. 5: i.e. without the additional changes associated with the condition being Smooth or Noisy (lower left panel of Fig. 5). Eleven simulations of pursuit are plotted, each with a value of ln *Π*_*s*_ varying between its values in the Smooth condition (thick red line) and Noisy condition (thick blue line). The target itself moved with the same (smooth) motion each time. Increasing ln *Π*_*s*_ has two obvious effects: when the target is visible, the eye tends to lag more behind the target (indicated with three asterisks), and when the target is occluded (between the grey bars), the size of the anticipatory saccadic movement is smaller.

The biggest difference between the two studies is that previously, target noise increased subjective precision at a higher level of the model (ln *Π*_*x*_); whereas no such effect was observed in this study. This is because – in the final quarter of the cycle – decreasing ln *Π*_*x*_ has the same effect as decreasing ln *Π*_*s*_. In other words, it increases the speed of lag correction (a simulation illustrating the effects of changing ln *Π*_*s*_ is shown in Inline Supplementary Fig. S2). In the previous study, lag was corrected more quickly in the final quarter in the Smooth condition (Fig. 6), hence in that study, target noise increased both ln *Π*_*s*_ and ln *Π*_*x*_.

Inline Supplementary Figure S2

Our first hypothesis was that target motion noise should increase the gain or excitability of superficial pyramidal cells at the sensory level (i.e. V1), reflecting an increase in sensory precision. Target noise reduce self inhibition in central and left peripheral V1 (cuneus), as we had anticipated. Interestingly, the opposite effect was observed in right peripheral V1, which reduced its gain. This asymmetrical pattern may be due to the fact that the eyes were on average ahead of the target (moving from right to left) when it emerged from the occluder (Fig. 5), thus the target would appear mainly in the right hemifield, encoded in left peripheral V1 (Fig. 8, right panel). We interpret this pattern of changes as indicating that increasing sensory attention does not merely correspond to a uniform increase in gain in primary visual areas, but instead an increase in gain in the specific part of V1 at which the signal is expected to arrive. This interpretation could be tested straightforwardly by repeating the paradigm with the horizontal axis reversed.

Target motion noise also led to decreases in forward connectivity from central V1 but increases from peripheral V1, especially on the right (Fig. 8, left panel), and reduction of synaptic gain in V2 bilaterally. We had no hypotheses relating to the connectivity between these areas, and so any interpretation of these findings must be speculative. It seems plausible, however, that the noisier target would elicit more activity in peripheral V1 and less in central V1 than the smoother target, and so, given forward connections carry prediction errors, it is not surprising that the connectivity between V1 and V2 changes likewise.

Finally, we hypothesized that patterns of precision changes in pursuit and MEG DCMs should correlate on an individual subject basis. We found a negative correlation between target noise-induced sensory precision changes and mean self-inhibition changes in V1. This is what one would expect if precision is encoded by the gain (disinhibition) of superficial pyramidal cell populations.

It should not be surprising that precision changes at the sensory level can be inferred from eye movements: over 90% of the variation in eye trajectory during pursuit initiation can be ascribed to variation in sensory estimation in both monkeys (Osborne et al., 2005) and humans (Mukherjee et al., 2015). Rather than looking at how errors in sensory estimates affect motor responses trial by trial, as Osborne and colleagues have done, we have shown that the average effect of noisy sensory input is to increase sensory precision (ln *Π*_*s*_) in the predictive coding of visual information. Simulations of pursuit using our generative model (Inline Supplementary Fig. S2) indicate that increasing ln *Π*_*s*_ results in a greater lag of the eye behind the target when it is visible — and a smaller anticipatory saccade when the target is occluded. This slightly counterintuitive finding – that increasing sensory precision (attention) can reduce pursuit velocity – is because the ‘pull’ of the attracting location is (relatively) diminished throughout the cycle. It is because of these effects of ln *Π*_*s*_ on the eye trajectory that the pursuit DCM can estimate ln *Π*_*s*_ from eye movements.

It is unfortunate that sources for FEF were not identified using source reconstruction, as including FEF would have given the DCM a more complete portfolio of cortical pursuit areas. Nevertheless, our hypothesis concerned the effects of target noise on sensory precision (i.e. V1/2) rather than higher level precisions, and so, from this perspective, omitting FEF from the DCM is of less importance.

Clearly, much of this discussion rests on assuming that we can interpret various levels of the pursuit model in terms of levels in the cortical hierarchy. We acknowledge that this assumption is tentative. In principle, the mapping between the computational model and the biophysical model would be best addressed using Bayesian model comparison and a single model that generated both (Bayes optimal) behaviour and electromagnetic responses.

More generally, this model has some key similarities with that of Orban de Xivry et al. (2013), who showed that important characteristics of both visually-guided and predictive pursuit movements can be reproduced by a model containing two Kalman filters: one processing (delayed) visual input and one dynamically updating an estimate of target motion. Both models use precision-weighted prediction errors (the Kalman gain depends on the precision of the errors) to update or integrate state estimates within generative models, highlighting the crucial use of uncertainty in (Bayes optimal) inference.

One difference between our approaches is that Orban de Xivry et al. (2013) use one Kalman filter to estimate the target's retinal slip from delayed sensory information, and another Kalman filter to predict the retinal slip 150 ms in the future (compensating for visuomotor delays) using an optimal estimate of target motion. Our model performs Bayesian filtering in generalised coordinates of motion, such that states (e.g. position) are represented along with their higher order derivatives (e.g. speed, acceleration, etc). This gives the model an implicit representation of the near past and future that can compensate for oculomotor delays by absorbing them into the model (Perrinet et al., 2014), and so both retinal input and eye movement can be predicted within one hierarchical model. Another major difference is that Orban de Xivry et al. (2013) focused on modelling trial-by trial pursuit data, whereas our original pursuit model (Adams et al., 2012) was adapted to model grand averaged eye trajectories that incorporate saccadic eye movements as well as pursuit, in order to estimate (average) sensory level precision from averaged eye trajectories (just as average synaptic gain can be estimated from averaged MEG data). Having noted the differences, it is interesting to note the convergence of recent modelling initiatives on Bayesian filtering (i.e., Kalman filtering or predictive coding) as a normative approach to pursuit eye movements. One might hope that the biological substrates of these theoretical perspectives can be clarified using procedures of the sort that we have described above.

On a practical note, in our dynamic causal modelling of MEG responses, we elected to identify the best model using grand average data — and then estimated subject-specific model parameters by inverting each subject's data under the best model. This contrasts with the alternative approach of inverting each subject and selecting the best model using (fixed or random effects) model comparison. We chose to perform Bayesian model comparison using the grand average for computational and statistical efficiency: computationally, this means we only have to invert one (grand average) dataset. Furthermore, inverting the grand average can sometimes finesse convergence and local minima problems encountered with inversion of individual subjects. This issue is discussed more fully in Friston et al. (2015), which shows that inversion of the grand average provides very similar estimates to the average of individual inversions; despite the fact that the two procedures are not equivalent (for nonlinear models).

## Conclusion

This study offers a construct validation of a generative model of pursuit that was designed to estimate subjective precisions from eye movements. More specifically, we have shown – using DCM to invert a generative model of oculomotor behaviour – that when tracking a sinusoidally moving target whose motion is noisy, subjects increase their sensory precision; i.e., they attend more to the sensory attributes of the target. Given that precision is thought to be encoded by the gain of superficial pyramidal cells (parameterized as disinhibition in DCM for MEG), we hypothesized that noisy target motion would disinhibit these connections in visual cortex. Using DCM of the visual response evoked by the target's emergence from an occluder, we found that there was indeed a robust reduction of self inhibition in central and left V1 when the target motion was noisy. Crucially, there was also a correlation (over subjects) between sensory precision estimated using DCM of pursuit behaviour, and the gain of superficial pyramidal cells in V1.

Note that in this study we are not seeking to validate our model of oculomotor pursuit: this model is a tool for us to validate our methods of inferring changes in subjective precision. We do not therefore compare the performance of our pursuit model with the many other well-established SPEM models in the literature (Barnes and Asselman, 1991, Krauzlis and Lisberger, 1989, Robinson et al., 1986, Shibata et al., 2005) for two reasons. First, our pursuit model treats SPEM and saccades as quantitative variants of the same process and is thus fundamentally different from traditional models: in short, it is not a model of SPEM per se, but a means of inferring subjective precision. Second, traditional models are not based upon hierarchical predictive coding and do not contain precision parameters (save perhaps for the ‘gain’ of the ‘retinal velocity error’ signal).

This is the first use of DCM to invert generative models of both behavioural and imaging data obtained concurrently. It is encouraging that predictions of one model are borne out in the other, but ultimately our aim is to integrate the generative models of behavioural and imaging responses. In other words, have a single bio-behavioural DCM that predicts both eye movements and neuronal responses. An important motivation for this work is that there are good theoretical reasons to suppose that the encoding of precision goes awry in many psychiatric disorders, particularly schizophrenia and autism (Adams et al., 2013b). We hope to use this paradigm in schizophrenic patients, to investigate whether we can infer the loss of prior (i.e. higher hierarchical) precision or increase in sensory precision that one might expect from their eye movements (Adams et al., 2012) and from other paradigms that demonstrate this precision imbalance in the disorder (Deserno et al., 2012, Dima et al., 2010, Hirano et al., 2015, Shergill et al., 2005). Correlations of behavioural estimates of key quantities (like sensory precision) with imaging findings at the individual subject level could pave the way towards new methods of phenotyping or diagnosing patients. If such assessments can be performed simply and inexpensively (e.g. tracking eye movements), all the better.

## Figures and Tables

**Table 1:** Prior expectations of model parameters and log precisions.

Parameter class	Model parameter	Short description	Prior expectation
Kinetic	(*θ*_1_, *θ*_4_)	Parameters encoding how gaze is attracted to the invisible location — occluder independent and dependent.	$\left(\frac{1}{4},\frac{1}{32}\right)$
(*θ*_3_, *θ*_5_)	Parameters encoding how gaze is attracted to the target location — occluder independent and dependent	$\left(\frac{1}{2},\frac{1}{32}\right)$
(*θ*_2_, *θ*_6_)	Parameters encoding the viscosity of eye and target motion (fixed between experimental conditions)	$\left(\frac{1}{2},\frac{1}{4}\right)$
Precision	ln *Π*_*s*_	Log precision of sensory noise	4
ln *Π*_*x*_	Log precision of eye and target motion	4
ln *Π*_*v*_	Log precision encoding the motion of the attracting location	4
Prior	(*θ*_7_, *θ*_8_)	Parameters encoding the amplitude and phase lag behind the invisible attracting location	$\left(1,\frac{2\pi }{32}\right)$

**Table 2:** Prior locations for sources used in the DCM analysis, defined using multiple sparse priors source localisation (see also Inline Supplementary Fig. S1). Prior locations for sources used in the DCM analysis, defined using multiple sparse priors source localisation (see also Inline Supplementary Fig. S1).

Source	MNI coordinates
V1	− 1 − 95 5
Cuneus L (peripheral V1)	− 10 − 79 28
Cuneus R (peripheral V1)	10 − 79 28
V2 L	− 24 − 94 − 19
V2 R	25 − 94 − 17
V5 L	− 46 − 74 12
V5 R	50 − 70 6
